# Supplementary material for: Comparative transcriptomics of Diuraphis noxia and Schizaphis graminum fed wheat plants containing different aphid-resistance genes
Source: PLoS One. 2020 May 22;15(5):e0233077. doi: 10.1371/journal.pone.0233077 (PMC7313535; doi:10.1371/journal.pone.0233077)
Supplement: Attachment — Submitted filename: Response to Reviewers.docx [file pone.0233077.s011.docx]

April 23, 2020

Owain Rhys Edwards, Ph.D.
Academic Editor
PLOS ONE

Dear Dr. Edwards,

Below please find author responses to reviewer and editorial comments regarding PONE-D-19-24276R1 “Comparative transcriptomics of *Diuraphis noxia* and *Schizaphis graminum* fed wheat plants containing different aphid-resistance genes.”

**Both reviewers have two related questions to be addressed:**

Q.1. Is Yuma an appropriate control plant for the 94M370 resistant line containing Dn7?

Q.2. Can the DEGs observed between 94M370 and Yuma reasonably be attributed to the Dn7 resistance gene?

Q.1. **Author’s Response.**  We agree with the reviewers that the use of appropriate controls is an important issue. The *Dn4* and *Dn7* genes have been previously linked to *D. noxia* resistance in a previous study using Gamtoos wheat as a control (Zaymaan et al. 2009 Physiol Plant 136: 209-222).  The authors elected not to use Gamtoos as a 94M370 control because the Gamtoos 1RS arm contains the rust resistance genes *Sr31* and *Lr26*, but the Turkey 77 wheat 1RS arm in 94M370 contains only *Dn7 (*reference 32 of current revision). The purpose of this current study was to determine whether two lines carrying two different resistance genes for *D. noxia* also had resistance to *S. graminum*.

We have shown that *Dn4* plants are susceptible to *S. graminum* and we have shown that the wheat variety 94M370 containing the *Dn7* gene displays some degree of resistance against *S. graminum.*  We suspect that this resistance could be conferred by *Dn7*, but it is possible that the genetic background of Gamtoos or a combination of the genetic backgrounds of 94M370 and Gamtoos could responsible for this resistance.  We have edited the manuscript to reflect this possibility (see response to Q2 below).

Since our objective was to compare transcriptional responses of *D. noxia* and *S. graminum* feeding on lines that had resistance to *D. noxia* only (Yumar) or both aphids (94M370) and to determine whether 94M370 plants had similar effects on gene expression in both aphid species, we believe that our differential expression analysis and comparisons presented in the results section are valid.  However, we now refer to *Dn7* as 94M370 to reflect the idea that differences in genetic backgrounds between *Dn0* and 94M370 could be responsible for some of the observed transcriptional effects observed in both aphid species.

**Q2. Author’s Response.** The reviewers and editor are correct that the DEGs observed between 94M370 and Yuma could be influenced by differences in genetic background in addition to the *D. noxia* *Dn7* resistance gene.  To address this concern, we have revised the manuscript in lines 92-98 and lines 885-862 to reflect this possibility.  In addition, we also refer to plants containing the *Dn7* gene as plants from wheat genotype 94M370 instead of *Dn7* plants as shown in the comment boxes throughout the Revised Manuscript with Track Changes file and the titles of several figures and Supplemental tables.  We offer the dataset in the current revision as a first attempt to understand Russian wheat aphid - greenbug comparative transcriptomics and refer to them as "likely species-specific changes," allowing readers to develop a broad interpretation of the results.

**Additional Editor Comment:** The authors may need to reconsider referring to the lines as "Dn0", "Dn4", and "Dn7" - as this may suggest to a reader who has not read the methods in detail that the hosts are all near-isogenic, differing only in the presence/absence of these genes. While this is the case for "Dn0" and "Dn4", it is definitely not the case for "Dn7".

**Author’s Response.** As indicated above, the revision includes reference to *Dn7* plants as 94M370 plants when discussing effects on *S. graminum* throughout the manuscript.

As requested, the following items have been included in the revision of PONE-D-19-24276_R1:

- A rebuttal letter responding to each point raised by the academic editor and reviewers uploaded as a file labeled “Response to Reviewers.”
- A marked-up copy of the second revision to the manuscript highlighting the changes in response to reviewer’s questions in PONE-D-19-24276_R1 uploaded as a file labeled “Revised Manuscript with Track Changes.”
- An unmarked version of the second revision manuscript without tracked changes uploaded as a file labeled “Manuscript.”

On behalf of all of the coauthors, we thank you very much for all of your assistance in the processing and handling of this manuscript.

Kind regards,

C. Michael Smith,

Fellow AAAS, ESA

University Distinguished Professor Emeritus
